# Chronic Pain and Sleep Disorders in Primary Care

**DOI:** 10.1155/2017/9081802

**Published:** 2017-12-19

**Authors:** Robert Jank, Alexander Gallee, Markus Boeckle, Sabine Fiegl, Christoph Pieh

**Affiliations:** ^1^Department of Psychotherapy and Biopsychosocial Health, Danube University Krems, Krems, Austria; ^2^Practice for General Medicine, Vorderweißenbach, Austria; ^3^Department of Psychosomatic Medicine, University Hospital Regensburg, Regensburg, Germany

## Abstract

**Background:**

Chronic pain (CP) and sleep disorders (SD) are highly prevalent in the general population. However, comprehensive data regarding the prevalence and characteristics of pain and SD in primary care are rare.

**Methods:**

From *N* = 578 patients *N* = 570 were included within 8 weeks (mean age: 50.8 ± 18.7 years, females: 289). Sociodemographic data, Insomnia Severity Index (ISI), and parts of a self-report questionnaire for pain (Multidimensional German Pain Questionnaire) were recorded and additional medical information (pain medication, sleep medication) was gathered from the patient charts.

**Results:**

Of the total sample, 33.2% (*n* = 189) suffer from CP (pain ≥ 6 months) and 29.1% (*n* = 166) from SD. 45.5% of the CP patients suffer from SD and 26.5% from clinical insomnia (ISI ≥ 15). SD (*β* = 0.872, SE = 0.191,  *t* = 4,572, *p* < 0.001, CI [0.497; 1.246]) and older age (*β* = 0.025, SE = 0.005, *t* = 5.135, *p* < 0.001, CI [0.015; 0.035]) were significantly associated with pain experience.

**Conclusion:**

About a quarter of CP patients suffer from clinical insomnia. The suggested bidirectional relation should be considered during comprehensive assessment and treatment of patients.

## 1. Introduction

Chronic pain (CP) and sleep disorders (SD) have become two of the most common symptoms reported by primary care patients. The prevalence of CP ranges from 10 to 40% [1], which is similar to prevalence rates of SD (10–36% [2–8]). CP is tied to considerable personal impairment in quality of life [9, 10]. Due to the high prevalence in the general population and its prominent disabling nature, CP imposes a burden, not only for the patient but also for the society in terms of economic productivity and health care costs [11–14]. The results of an European cross-sectional survey report that 19% of CP patients lost their job and 13% had to change their jobs [15]. These indirect costs (informal caregiving, work impairment, etc.) cause significantly higher expenditures than direct health care costs alone [16]. Comparable to patients with CP, patients with SD suffer from poor quality of life and considerable health restrictions and social limitations [17, 18]. SD are well-documented risk factors for psychiatric diseases [19–21], cardiovascular diseases [22, 23], and increased mortality [24].

There is limited but growing epidemiological information on the prevalence of CP and comorbid SD in primary care. In clinical settings SD have been found to impact 50–88% of CP patients [25, 26]. Vice versa, more than 40% of patients suffering from SD report CP [4]. Cooccurring health problems are considered as the norm rather than the exception in primary care patients [27]. They increase the risk of premature death, hospitalization, loss of physical functioning, depression, poly-pharmacy, reduced quality of life, and increased health care costs [28]. The interaction of sleep and pain is complex and still not well understood [7]. A recent meta-analysis confirms a medium effect of sleep deprivation on pain perception in healthy individuals [29]. However, the occurrence and size of this effect are unclear in clinical relevant populations. There is increasing evidence suggesting a reciprocal relationship between pain and sleep. Results from studies in animal models [30–33], healthy persons [5, 34–37], and patients with different types of pain [38–42] support the reciprocal interaction of CP and SD. For example, pharmaceutical studies on eszopiclone [43, 44], triazolam [45], or pregabalin [46] show a simultaneous improvement of both pain and sleep.

The presence of comorbid SD in patients with diabetic peripheral neuropathy and postherpetic neuralgia may even predict substantial pain relief in response to pain treatment with pregabalin [47].

Patients with several SD, such as obstructive sleep apnea syndrome (OSAS) [48] and restless legs syndrome (RLS) [49], seem to be hyperalgesic. Effective treatment of the respective sleep disorder improves pain sensitivity. This improvement has been demonstrated by pharmacological interventions against pain, such as levodopa (L-DOPA) therapy for RLS [49], and in nonpharmacological interventions, such as continuous positive airway pressure (CPAP) treatment for OSAS [48]. Despite the suspected high prevalence of SD in the general population, they are often underidentified by primary care professionals and thus a large percentage of patients remain untreated [18]. Only in about half of the cases the treating general practitioner knows about sleeping problems [50]. Given the significant impact of SD on pain experience and the fact that pain is a frequent reason for primary care consultations, the focus of this study was to explore the prevalence and characteristics of CP and SD in primary care patients.

## 2. Methods

### 2.1. Participants

Participants were patients of a general practice in Austria. In this practice, patients are treated by a licensed general physician who underwent a qualification programme for psychosomatic and psychotherapeutic medicine (including, e.g., sleep and pain) at the time of the study. The practice is the only general practice for a relatively large rural region where mainly workers and agriculturalists live.

Within eight-week duration (06/01 to 07/24 2015) every patient of the general practice was asked to participate. The study nurses screened all potential participants for their eligibility and their level of cooperation in order to raise the responder rate. Subjects who were unable to complete the questionnaires due to linguistic barriers or serious physical or psychological health issues and patients younger than 10 years were excluded. After the initial information session all subjects were provided with questionnaires in the doctor's waiting room prior to the consultation. The following questions regarding pain and sleep disorders were included: “Do you suffer from pain/sleep disorders?” If this was answered by “yes” subjects were asked to proceed answering the relevant questions. In case patients did not know how to answer a question, the study nurses gave explanations.

### 2.2. Measures

#### 2.2.1. Pain

Pain was assessed according to the Multidimensional German Pain Questionnaire (Deutscher Schmerzfragebogen (DSF) [51]). The items comprised type of pain (“do you have background pain, background pain with breakthrough pain, breakthrough pain?”), duration (“since when do you have pain?”), and localization (“where on your body do you feel pain?”). CP was defined by pain ≥ 6 months. The instrument is empirically evaluated [52] and also includes questions concerning age, gender, weight, and height.

#### 2.2.2. Education

Self-reported level of education was assessed by using 4 response options. We asked for the highest educational attainment. Employment status was defined by using a 3-point categorical variable. Job seekers, person doing family work, and retired persons were assigned as “unemployed.” We categorized worker, piece worker, employees, self-employed people, apprentices, volunteers, and internships as “employed.” Scholars and students were categorized as “in education.” The specific categories of education as well as the ones of employment status can be seen in Table 1.

#### 2.2.3. Medical Information

The current use of prescribed pain and sleep medication was gathered from the patients' charts. The coding of the medication as medication for pain or medication for sleep was done by A.G. According to the “Pain ladder," or analgesic ladder, created by the World Health Organization (WHO) [53], nonopioids (nonsteroidal anti-inflammatory drugs) and weak opioids were coded as pain medication. Non-benzodiazepine hypnotics (Z-Drugs), benzodiazepines, and sedating antidepressants (off-label sleep aids) were coded as sleep medication. If patients were prescribed sleep medication and pain medication together, we coded as “combinations.”

#### 2.2.4. Sleep Disorders

SD were assessed by the Insomnia Severity Index (ISI), a brief self-report questionnaire consisting of seven items. Each item has to be rated on a five-point Likert scale, ranging from 0 (not at all) to 4 (extremely). The items are problems initiating sleep, problems maintaining sleep, early awake, satisfaction with current sleep patterns, impact on daytime quality of life due to SD, noticeability of sleep problems by others, distress or worry caused by SD, and interference with daytime functioning. Total scores range from 0 to 28. Higher scores indicate a greater insomnia severity (0–7: no clinically significant insomnia; 8–14: subthreshold insomnia; ≥15: clinical insomnia). The psychometric properties are considered to be sound [54, 55].

### 2.3. Source of Funding and Ethical Considerations

The study was conducted in accordance with the Declaration of Helsinki and approved by the local ethical committee of the Danube University of Krems.

Patients were informed about the voluntary nature of participation and informed consent was obtained from each subject. If participants were younger than 18 years also parents had to agree. This study was conducted independently of any institutional influence and was not funded externally.

### 2.4. Statistics

The statistical analyses were performed with SPSS 23.0. Frequencies (*N*, *n*), percentages (%), means (M), and standard deviations (Std) were calculated as descriptive statistics; Chi-squared tests were performed to compare men and women. Individuals were excluded from the statistical analysis of a variable if they had a missing value in this given variable.

We modeled predictors for pain experience and sleep disturbance with type III generalized linear models (GLM). We used standard stepwise backward model selection procedures based on AICc [56] for identification of predictor variables. The backward method and not the forward method was selected because the forward method has a higher Type II error risk [57].

## 3. Results

### 3.1. Sample

A total of *N* = 570 (*N* = 289 female) patients participated in the study. This equals a response rate of 98.6%. The mean (Std) age was 50.8 (±18.7), for women 50.8 (±18.8) and for men 50.9 (±18.6) years. The majority attended compulsory school and apprenticeship (80.2% of the total sample). About half of the patients were employed (53.7% of total sample). The mean of the body mass index in both genders is slightly increased. Not medicated were 67.9%, 22.6% took medications for pain, 5.4% received medication for sleep, and 3.2% were medicated for both pain and sleep. Further results for pain are summarized in Table 2. Detailed data are presented in Table 1.

### 3.2. Clinical Variables

#### 3.2.1. Pain

Of the total sample *n* = 238 (41.8%) answered yes to the question “do you suffer from pain?” indicating pain experiences (41.3% of the men versus 42.2% of the women; *p* = 0.82). Pain duration of at least 6 months (CP) was reported by *n* = 189 (33.2% of total sample; see Table 2).

#### 3.2.2. Sleep

One hundred and sixty-six patients of the total sample (29.1%) answered yes to the question “do you suffer from sleep disorders?” (25.6% of the men versus 32.5% of the women; *p* = 0.04) and *n* = 33 (5.8%) of the total sample suffered from a clinical insomnia (see Table 3). Of the *n* = 189 patients with CP, *n* = 86 patients (45.5%) also reported SD. Of the *n* = 86 patients with CP and SD, *n* = 83 also completed the ISI and *n* = 22 (26.5% of *n* = 83) had a clinical insomnia (ISI ≥ 15). Table 3 provides an overview for the patients with self-reported sleep disorders.

### 3.3. General Linear Model

We include 567 individuals in the full model. The full model includes age, gender, BMI, employment status, education and sleep disturbances as correlates of experience of pain. After the stepwise reduction of variables the final model includes age (*F*1, 567 = 29.123, *p* < 0.001) and SD (*F*2, 567 = 20.903, *p* < 0.001). SD was significantly associated with pain experiences (*β* = 0.872, SE = 0.191,  *t*= 4,572, *p* < 0.001, CI [0.497; 1.246]).

Older age was associated with pain experience also significantly (*β* = 0.025, SE = 0.005, *t* = 5.135, *p* < 0.001, CI [0.015; 0.035.]).

## 4. Discussion

This study underlines the high prevalence of CP and SD in primary care. 33.2. % suffered from CP. Out of all participants 29.1% reported SD. 26.5% of CP patients suffered from clinical insomnia. These findings confirm results from previous studies [4, 36]. In contrast, several studies indicate a higher rate of SD in pain patients [40, 58]. These differences can be partially explained by the different types of pain (pain experienced in the last four weeks versus CP). This could be supported by Fishbain and colleagues [59], who showed that SD are significantly more prevalent in CP patients than in patients with acute pain.

In our study SD and older age were significantly associated with pain as already reported [60]. Employment status and education were not significantly correlated with pain. These results are not in line with earlier studies [61–63]. One possible reason might be the sample characteristics covered by the study. Our participants showed lower education attainments [64] and a higher unemployment rate [65] than Austria as a whole.

We found a higher prevalence in patients suffering from initiating sleep, maintaining sleep, early awake, and dissatisfaction with sleep in comparison to Baker et al. [60]. The most likely reasons for these differences might be that we considered longer durations of pain.

The results did not confirm existing evidence, that women experience chronic pain more often than men [66, 67]. As a recent systematic review and meta-analysis revealed, chronic pain prevalence estimations are an obvious matter of survey methods [68]. Our defining of CP (≥6 month) and data collection method might explain these differences. We found no differences in the prevalence of pain type by gender [69]. Male and female patients suffered around the same frequency from background pain, background pain with breakthrough pain, and breakthrough pain. We found gender differences in pain sites. The most common body sites in women affected by pain were 1st multiple sites, 2nd joint pain, 3rd back pain, 4th headache, and 5th chest pain. Men reported most frequent 1st multiple site pain, 2nd back pain, 3rd joints pain, 4th chest pain, 5th headache, and 6th abdominal pain. These results confirm findings from a recent Austrian study [62] that found comparable frequencies. Our result revealed that headache is significantly more common in women [62, 67].

SD are often overlooked in CP patients [70]. This is especially intriguing as treating cooccurring SD has a high potential to improve pain management outcomes [71]. About half of CP patients with SD in our study were prescribed with pain medication, about 10% to 20% with sleep medication or combinations of both. Female patients received more often sleep medications or both, sleep and pain medications. In clinical practice it would be beneficial to pharmacologically treat CP and SD jointly [7, 72]. There are several pharmacological approaches that can improve both CP and cooccurring SD. Some tricyclic antidepressants (amitriptyline, nortriptyline, trimipramine, and doxepin) show positive effects on both, CP and SD [16, 35, 40, 73, 74]. Trazodone, a serotonin modulator, has been studied in patients with various types of pain. The outcome was associated with both improved sleep quality and pain reduction [25]. Mirtazapine is effective in the treatment of SD [32] as well as for the treatment of pain [15, 71].

Gabapentin and pregabalin are also often used to treat CP and cooccurring SD [60, 75, 76]. Short term use of benzodiazepines (BRA) [45, 75, 77] and non-benzodiazepine drugs (NBRA) [43, 44, 78] has been shown to be potentially useful in improving SD in CP patients. In contrast to benzodiazepines [74], long- term efficacy trials have supported the use of NBRA [76, 79, 80].

A variety of psychological interventions have been found to be effective in both pain treatment [81–84] and insomnia treatment [73, 85]. Psychological techniques for CP management usually involve strategies for the identification of dysfunctional and/or maladaptive thoughts and specific strategies, such as distraction techniques, relaxation training, and activity pacing. These skills help patients to manage pain symptoms [70]. Psychological interventions for SD typically utilize behavioral interventions like general sleep education, stimulus control instructions, sleep hygiene education, alternation of biorhythm, and sleep restriction [71]. Given the effectiveness of these interventions, there has been growing interest in simultaneous treatments of the symptoms. Combined treatments utilize components of both interventions. In a pilot study with CP patients, Tang and colleagues [86] found a greater improvement in sleep after treatment, in comparison to a waiting group, who kept pain and sleep diaries.

The results of our study have to be interpreted with respect to its limitations. The generalizability of the results is limited as only one general practice took part in the study. Yet, some aspects speak for the generalizability of the results. First, patients were treated under the conditions of routine. Second, the practice is the only general practice in a relatively large rural region. Moreover, it is important to note that the sample is based on a cross-sectional design, which does not allow causal inferences.

Additionally, we did not assess psychiatric conditions, which are frequently associated with SD and pain [87, 88]. Another limitation is that we did not carry out comprehensive assessment of pain and sleep disorders. For example, the screening question “do you suffer from pain/sleep disorders?” was not validated before and it is not clear how children understood the questions. However, only two participants were younger than 15 years and it has been shown that even children five years old can give meaningful self-reports of pain when age-appropriate tools and training are provided [89]. While the present study has limitations, the results are based on a relatively large sample size with an extraordinary high response rate. Thus we can provide an almost complete evaluation of the cooccurrence of SD and pain experiences in patients of a general practice.

Comprehensive management of CP and SD in primary care requires an individualized assessment and treatment approach. A shift from predominantly biomedical strategies to a more biopsychosocial perspective would raise treatment outcomes. SD should be systematically evaluated in CP patients in primary care settings based on the reported high cooccurrence. A proactive diagnostic approach for these persons is needed to achieve accurate information and treatment. Coordinated multidisciplinary and interdisciplinary care has a high potential to provide this. The high influence on personal life-quality and health care costs make it important that increased efforts in experimental and clinical research are made. The aim of these efforts should be a translationally meaningful understanding of the mutual impact of CP and poor sleep.

## Figures and Tables

**Table 1:** Sociodemographic data, body mass index, and pain and sleep medication *n* (%).

	Male	Female
*Gender n* (%)	281 (49.3)	289 (50.7)
*Age* M years (±Std^*∗*^)	50.9 (18.6)	50.8 (18.8)
*Education* % (*n*)^#^		
Compulsory school 9 years *n* = 181	63 (34.8)	118 (65.2)
Apprenticeship Compulsory school and additional training of at least 2 years in a specific occupational scope *n* = 276	162 (58.7)	114 (41.3)
Matriculation equal a higher school graduation *n* = 60	31 (51.7)	29 (48.3)
University, University of Applied Science *n* = 51	24 (47.1)	27 (52.9)
*Employment status n* (%)^+^		
Unemployed/retired *n* = 209	109 (52.2)	100 (47.8)
Employed *n* = 306	146 (47.7)	160 (52.3)
In education *n* = 45	23 (51.1)	22 (48.9)
*BMI* Mean (±Std^*∗*^) *n* = 568	27.1 (4.7)	26.2 (5.3)
*Medication n* (%)^−^		
None *n* = 387	201 (51.9)	186 (48.1)
Pain *n* = 129	61 (47.3)	68 (52.7)
Sleep *n* = 31	11 (35.5)	20 (64.5)
Combinations *n* = 18	5 (27.8)	13 (72.2)

^*∗*^Standard deviation (Std). ^#^Education was available for *n* = 568 patients (99.6% of total sample). ^+^Employment status was available for *n* = 560 patients (98.2% of total sample). ^−^Medication was available for *n* = 565 patients (99.1% of total sample).

**Table 2:** Pain.

	% of total sample (*N* = 570)	% of sample with pain experiences (*n* = 238)	Male (*n*, %% of the *n* given in the left column)	Female (*n*, %% of the *n* given in the left column)	*p* value for the comparison men versus women
*Type of pain* ^*∗*^					
Background pain *n* = 85	14.9	37.6	40 (47.1)	45 (52.9)	*p* = 0.89
Background pain with breakthrough pain *n* = 17	3.0	7.5	9 (52.9)	8 (47.1)	
Breakthrough pain *n* = 124	21.8	54.9	61 (49.2)	63 (50.8)	

*Duration of pain* ^#^					
Less than 1 month *n* = 17	3.0	7.2	13 (76.5)	4 (23.5)	*p* = 0.31
One to six months *n* = 29	5.1	12.3	13 (44.8)	16 (55.2)	
Six to twelve months *n* = 12	2.1	5.2	6 (50.0)	6 (50.0)	
One to two years *n* = 32	5.6	13.6	16 (50.0)	16 (50.0)	
Two to five years *n* = 41	7.2	17.4	20 (48.8)	21 (51.2)	
More than 5 years *n* = 104	18.2	44.3	47 (45.2)	57 (54.8)	

*Localization of pain* ^+^					
Head *n* = 18	3.2	7.9	4 (22.2)	14 (77.8)	*p* = 0.02
Chest *n* = 6	1.1	2.6	5 (83.3)	1 (16.7)	
Abdominal *n* = 2	0.4	0.9	2 (100.0)	0 (0)	
Back *n* = 51	8.9	22.5	30 (58.8)	21 (41.2)	
Joints *n* = 56	9.8	24.7	29 (51.8)	27 (48.2)	
Multiple *n* = 94	16.5	41.4	42 (44.7)	52 (55.3)	

^*∗*^Type of pain was available for *n* = 226 patients (95.0% of the *n* = 238 patients with pain experiences). ^#^Duration of pain was available for *n* = 235 patients (98.7% of the *n* = 238 patients with pain experiences). ^+^Localization of pain was available for *n* = 227 patients (95.4% of the *n* = 238 patients with pain experiences).

**Table 3:** Sleep.

	% of total sample (*N* = 570)	% of sample with sleep disorders (*n* = 166)	Male (*n*, % of the *n* given in the left column)	Female (*n*, % of the *n* given in the left column)	*p* value for the comparison men versus women
CP and SD *n* = 86	15.1	51.8	39 (45.3)	47 (54.7)	*p* = 0.33

CP, SD, and problems falling asleep (ISI item 1 answered as mild, moderate, severe, or very severe) *n* = 64	11.2	38.6	27 (42.2)	37 (57.8)	*p* = 0.18

CP, SD, and problems maintaining sleep (ISI item 2 answered as mild, moderate, severe, or very severe) *n* = 66	11.6	39.8	30 (45.5)	36 (54.5)	*p* = 0.49

CP, SD, and early awaking (ISI item 3 answered as mild, moderate, severe, or very severe) *n* = 52	9.1	31.3	25 (48.1)	27 (51.9)	*p* = 0.85

CP, SD, and sleep dissatisfaction (ISI item 4 answered as dissatisfied and very dissatisfied) *n* = 31	5.4	18.7	13 (41.9)	18 (58.1)	*p* = 0.43

CP, SD, and impact of sleep disorder on performance (ISI item 7 answered as a little, somewhat, much, very much) *n* = 76	13.3	45.8	34 (44.7)	42 (55.3)	*p* = 0.34

CP, SD, and impact on other life areas (ISI item 5 answered as a little, somewhat, much, very much) *n* = 68	11.9	41.0	32 (47.1)	36 (52.9)	*p* = 0.69

CP, SD, and worries (ISI item 6 answered as a little, somewhat, much, very much) *n* = 62	10.9	37.3	32 (51.6)	30 (48.4)	*p* = 0.79

Clinical insomnia (ISI ≥ 15) *n* = 33^#^	5.8	20.8	16 (48.5)	17 (51.5)	*p* = 0.41

CP and Clinical insomnia (ISI ≥ 15) *n* = 22	3.9	13.3.	11 (50.0)	11 (50.0)	*p* = 0.72

^#^ISI score was available for *n* = 159 of the *n* = 166 patients with sleep disorders.
